# Side Effects of Curcumin: Epigenetic and Antiproliferative Implications for Normal Dermal Fibroblast and Breast Cancer Cells

**DOI:** 10.3390/antiox8090382

**Published:** 2019-09-09

**Authors:** Laura Cianfruglia, Cristina Minnelli, Emiliano Laudadio, Andrea Scirè, Tatiana Armeni

**Affiliations:** 1 Dipartimento di Scienze Cliniche Specialistiche ed Odontostomatologiche-Sez. Biochimica, Biologia e Fisica, Università Politecnica delle Marche, 60131 Ancona, Italy; 2 Dipartimento di Scienze della Vita e dell’Ambiente, Università Politecnica delle Marche, 60131 Ancona, Italy; 3 Dipartimento S.I.M.A.U., Università Politecnica delle Marche, via Brecce Bianche, 60131 Ancona, Italy

**Keywords:** curcumin, glutathione, post-translational modifications (PTMs), histone glutathionylation, human cancer cells

## Abstract

Background: Curcumin is a yellow-orange pigment obtained from the plant *Curcuma longa*, which is known to exert beneficial effects in several diseases, including cancer. However, at high doses, it may produce toxic and carcinogenic effects in normal cells. In this context, we studied the effects of curcumin on normal human dermal fibroblast (HDF) cells and breast cancer cells (MCF7). Methods: We used cellular viability and growth assays to evaluate the antiproliferative action of curcumin, analyzed the endogenous glutathione levels, conducted cell cycle, apoptosis, and necrosis analyses, and performed immunodetection of glutathionylated and acetylated H3 histones. Results: We found that HDFs are more sensitive to curcumin treatment than MCF7 cells, resulting in pronounced arrest of cell cycle progression and higher levels of cellular death. In both cell types, the homeostasis of the redox cellular environment did not change after curcumin treatment; however, significant differences were observed in glutathione (GSH) levels and in S-glutathionylation of H3 histones. Conclusion: Curcumin administration can potentially confer benefits, but high doses may be toxic. Thus, its use as a dietary supplement or in cancer therapies has a double edge.

## 1. Introduction

Curcumin (1,7-bis(4-hydroxy-3-methoxyphenyl)-1,6-heptadiene-3,5-dione) is a well-known dietary pigment and the main active component of the spice turmeric (*Curcuma longa*). *Curcuma longa* is a popular herb that is used in Ayurvedic medicine due to its therapeutic properties, which include analgesic, anti-inflammatory, and antiseptic activities. However, over the last few years, its major active constituent, the hydrophobic and polyphenolic compound curcumin, has received interest from the medical/scientific community. Indeed, many studies have shown that curcumin exerts antioxidant, anti-inflammatory, antibacterial, antifungal, antiproliferative, and antitumor activities [1,2,3,4]. In particular, curcumin exerts its anticancer activity through its ability to suppress cell proliferation, induce apoptosis, inhibit angiogenesis, and suppress the expression of antiapoptotic proteins [5,6,7,8]. Curcumin’s anticancer activity has been highlighted in several studies, both in vitro, where it induced apoptosis in cells of different cancer types, and in vivo, where it exhibited an antitumor effect in people with a precancerous lesion [9]. Moreover, it is known that the cells of several cancers are more sensitive to curcumin treatment than normal cells, which confirms its potential in cancer prevention and therapy [4]. However, accumulating evidence indicates that under specific conditions, curcumin may produce toxic and carcinogenic effects in nontumor cells, and this collateral effect should be carefully considered in pharmacological studies. Indeed, several reports have shown that curcumin can induce DNA damage in cells of several lines, including mammalian cells [10,11], human gastric mucosa (GM) cells [12], human peripheral blood lymphocytes [12], and bone marrow cells [10], both *in vitro* and *in vivo*, at concentrations similar to those that have been reported to exert beneficial effects (5–50 µM) [7,13,14]. The induction of alterations in DNA is a common event in carcinogenesis, and this clastogenic effect of curcumin has been shown *in vivo* to promote the development of lung cancer in mice [15]. In bone marrow cells of acutely treated mice [16] and in different tissues (e.g., liver and kidney) of male rats, a dose-dependent increase in the number of micronucleated polychromatic erythrocytes (MNPCEs) and the frequency of total chromosomal aberration was observed after curcumin treatment [17]. Besides this, some authors have demonstrated the ability of curcumin to influence cell cycle progression in normal oocytes [18] and induce apoptosis of normal resting human T cells [19]. Based on these results, we hypothesize that the effects of curcumin are cell type specific.

In this context, we explored the homeostasis of the redox cellular environment by measuring the glutathione level in a breast cancer-originating cell line and normal human fibroblasts and investigated the ability of curcumin to induce post-translational modifications (PTMs) in histones. We considered two modifications that are known to play specific roles in regulation of the chromatin structure and, consequently, gene transcription: acetylation and glutathionylation of the H3 histone, which is a redox-dependent PTM.

Moreover, our work showed that a dose-dependent curcumin treatment inhibits cellular proliferation in a breast cancer cell line (MCF7) and normal human dermal fibroblasts (HDFs), which were used as a control. Curcumin has been reported to have high cytotoxicity in cell cultures of fibroblasts [20] and after topical administration [21]; however, the mechanisms underlying this antiproliferative effect have not been fully investigated. For this reason, our experiments were directed to the cell cycle, cell apoptosis and necrosis, endogenous glutathione levels, and PTMs of H3 histones.

## 2. Materials and Methods

### 2.1. Reagents

Cell culture reagents and Enhanced Chemiluminescence (ECL) LiteAblot were obtained from Euroclone (Milan, Italy). Chemical reagents and secondary antibodies were obtained from Sigma-Aldrich (St. Louis, MO, USA). Carboxy-H2DCFDA (C400) was obtained from Invitrogen (Carlsbad, CA, USA). A histone H3 acetylation kit was purchased from Abcam (Cambridge, UK). An Annexin V-FITC Apoptosis Detection kit was obtained from Biolegend (San Diego, CA, USA). Mouse monoclonal anti-glutathione antibody was obtained from ViroGen (Watertown, MA, USA). Bradford reagent and polyvinylidene difluoride (PVDF) membranes were obtained from Bio-Rad (Hercules, CA, USA).

### 2.2. Cell Culture and Curcumin Treatment

Primary Human Dermal Fibroblast (Normal HDFa) (ATCC^®^ PCS-201-012™) and MCF7 (ECACC 86012803) cells were purchased from the American Type Culture Collection (ATCC, Italy office, Sesto San Giovanni, MI Italy) and the European Collection of Cell Cultures (ECACC), respectively. Cell lines were grown in Dulbecco’s modified Eagle’s medium (D-MEM) supplemented with 10% (v/v) heat-inactivated fetal bovine serum (FBS), 100 U/mL penicillin, and 2 mM glutamine at 37 °C in 5% CO_2_ and 95% humidity. For the curcumin treatment, cells were subcultured in six-well plates or in 12-well plates at a concentration of 12 × 10^4^ (six-well plates) or 8 × 10^4^ (12-well plates) and 1 × 10^5^ (six-well plates) or 7 × 10^4^ (12-well plates) for HDF and MCF7, respectively, and then incubated with 10 µM curcumin for 24 h. Curcumin was dissolved in DMSO, so control cells were cultured in a medium containing an equal amount of DMSO without curcumin. After incubation, cells were harvested and analyzed.

### 2.3. Cell Growth and Viability (MTT Assay)

Cell growth was determined in HDF and MCF7 cells after curcumin treatment by counting cell numbers in a hemocytometer. HDF and MCF7 cells were seeded in 12-well plates, treated with 5, 10, and 20 µM curcumin and counted at 24, 48, and 72 h after treatment. The viability of the cells was estimated by examining their ability to exclude Trypan blue (0.1% in 0.9% NaCl). The cell population doubling time was calculated using data that were extrapolated from the growth curves using the formula:

Doubling time = (t2 − t1) × ln2/ln (Nt/N0)
(1)

where N0 is the number of plated cells, Nt is the number of harvested cells, and t is the culture time in hours [22].

To determine the number of metabolically active cells, and thus the cell viability, we used the 3-(4,5-dimethylthiazol-2-yl)-2,5-diphenyltetrazolium bromide (MTT) assay [23]. At the time of analysis, the medium from each well was removed and replaced with fresh medium supplemented with 100 μg of MTT (50 μL from the 2 mg mL^−1^ stock); samples were incubated for 3 h at 37 °C in a 5% CO_2_ atmosphere until formazan crystals were formed. Next, 400 μL of DMSO was added to each well and mixed thoroughly by shaking to solubilize the MTT formazan crystals. Absorbance was read on a multiwell scanning microplate reader (the BioTek Synergy HT MicroPlate Reader Spectrophotometer) at 570 nm using the extraction buffer as a blank. The optical density in the control group (untreated cells) was considered as 100% viability. The relative cell viability (%) was calculated as (A570 of treated samples/A570 of untreated samples) × 100. Each experiment was performed at least five times in triplicate.

### 2.4. Cell Cycle Analysis

The cell cycle was analyzed using a Guava easyCyte flow cytometer (Millipore), and all measurements were performed with the same instrument setting. DNA content analysis was carried out using the propidium iodide (PI) method. Cells were harvested and washed twice with cold phosphate-buffered saline (PBS) then fixed with 70% ice-cold ethanol overnight at −20 °C for resolving the sub-G1 peak. The fixed cells were suspended in a master mix solution containing PBS, Propidium Iodide (final concentration: 40 µg/mL), and DNase-free RNaseA (final concentration: 100 µg/mL) for 30 min at room temperature in the dark. At least 5000 cells for each sample were measured. The percentage of cell cycle phases (G0/G1, S, and G2/M) was quantified using FlowJo^®^ software (FlowJo LLC, Ashland, OR, USA).

### 2.5. Apoptosis Analysis

Apoptosis was analyzed by flow cytometry using an Annexin V-FITC Apoptosis Detection kit in accordance with the manufacturer’s instructions. Briefly, cells were trypsinized, washed twice with cold PBS, and resuspended in 1X Binding Buffer at a final concentration of 10^6^ cells/mL. Annexin V-FITC (0.25 µg/mL) and propidium iodide (PI) (1 µg/mL) were added to the cell suspension and incubated for 10 min at room temperature in the dark. Samples were analyzed using a Guava easyCyte flow cytometer. For each sample, 5000 events were acquired. Annexin V-FITC is detected as a green fluorescence and propidium iodide is detected as a red fluorescence. Early apoptosis is defined by Annexin V^+^/PI^−^ staining, late apoptosis is defined by Annexin V^+^/PI^+^ staining, and necrosis is defined by Annexin V^−^/PI^+^ staining.

### 2.6. Histone Acetylation

Histone Acetylation was performed using an Acetylation Assay Kit (Abcam) in accordance with the manufacturer’s instructions. Briefly, cells were pelleted, resuspended in 1X Lysis Buffer, and incubated for 5 min at 4 °C. Pellet debris were obtained by centrifugation at 12,000× *g* for 1 min. Lysis Buffer (1X) was added to the cell debris, followed by three volumes of extraction/glycerol solution. The nucleic pellet debris and 100% 2,2,2-trichloroacetic acid (TCA) were added to the supernatant at a ratio of 1:4. The pellet was washed twice with acetone and incubated on ice for 1 min. Afterward, the pellet was collected by centrifugation, the supernatant was removed, and the pellet was air-dried. The pellet contained the histone fraction and was dissolved in ultrapure water. Then, the protein concentration was measured. The histone fraction was analyzed by ELISA. The H3 histone acetylation was calculated as:

Acetylation % = [OD _treated sample_ ‒ OD _blank_/OD _untreated_ ‒ OD _blank_] × 100 (2)

The acetylation of untreated cells was considered as 100% acetylation.

### 2.7. Immunoblotting

For immunoblotting, cell samples were washed twice with ice-cold PBS and lysed with cytosolic lysis buffer (50 mM Tris-HCl pH 7.2, 137.5 mM NaCl, 5 mM, Ethylenediaminetetraacetic acid (EDTA), 10% Glycerol, and a protease inhibitor cocktail) for 20 min at 4 °C. After incubation, the cells were treated with 0.5% (v/v) Triton X100 detergent and vortexed. The cells were then centrifuged at 10,000× *g* for 5 min at 4 °C. The supernatant was recovered, and this fraction contained the cytosolic extract. The pellet was resuspended in nuclear buffer (50 mM Tris-HCl pH 8.0, 150 mM NaCl, 0.1% (v/v), sodium dodecyl sulfate (SDS), 1% (v/v) Triton X100, and a protease inhibitor cocktail) and incubated for 30 min on ice. Nuclei were centrifuged at 16,000× *g* for 10 min at 4 °C. Nuclear extracts were recovered, and the protein content was determined by the method of Bradford [24] using bovine serum albumin (BSA) as a standard at an excitation wavelength of 595 nm. The histone fraction was obtained as described above.

Histone extracts (4 μg) were electrophoresed on a 16% acrylamide/bis acrylamide gel at 30 mA for 90 min. The analysis was carried out under nonreducing conditions in order to avoid the reduction of penicillamine-glutathione mixed disulfide PSSG derivatives. Proteins were transferred to an activated PVDF membrane at 100 V for 75 min. Histone S-glutathionylation was analyzed with a specific mouse monoclonal primary antibody that recognizes glutathione (GSH)-protein complexes (ViroGen, Watertown, MA, USA). To determine total H3 histone, nuclear extracts (30 μg) were electrophoresed on a 12% acrylamide/bis acrylamide gel at 30 mA for 90 min. After proteins were blotted on an activated PVDF membrane, the H3 histone was incubated with rabbit polyclonal anti-H3 antibody (#9715; Cell Signaling Technology, Inc. Danvers, MA, USA). Lamin B1 (D4Q4Z) Rabbit mAb (#12586; Cell Signaling Technology, Inc. Danvers, MA, USA) has been used as loading control. Goat anti-mouse and goat anti-rabbit secondary antibodies HRP (Horseradish Peroxidase) were used in accordance with the manufacturer’s instructions. Immunoblots were detected by enhanced chemiluminescence (ECL) (Euroclone Spa).

### 2.8. Glutathione Assay

Total glutathione (GSH + GSSG) and oxidized glutathione (GSSG) were measured spectrophotometrically (at 412 nm) using the glutathione reductase (GR) recycling assay in the presence of 5,5′-dithiobis (2-nitrobenzoic acid) (DTNB), with a calibration line that was based upon known concentrations of GSH and GSSG [25]. Cells were then trypsinized and washed twice in cold PBS for total GSH/GSSG determination. Then, the pellet was resuspended in 1% sulfosalicylic acid, vortexed, and incubated for 30 min at 4 °C. Samples were then centrifuged at 2300× *g* for 2 min, and the supernatant was separated into two aliquots: one was maintained at 4 °C for GSH quantification, and the other was treated with 2-vinilpiridin (Cf = 5%) and 20% v/v triethanolamine (Cf = 1%) to mask the GSH present in the extract and prevent its measurement. Finally, the pellet was resuspended in 1 M NaOH for the recovery and quantification of proteins [26].

### 2.9. Statistical Analyses

Data are presented as means ± SD. Statistical comparison of differences among groups of data was carried out using Student’s *t*-test. *p* values ≤ 0.05 were considered statistically significant, *p*-values ≤ 0.01 and *p*-values ≤ 0.001 were considered highly significant.

## 3. Results and Discussion

### 3.1. Curcumin Inhibits Cell Viability and Cell Growth

**Figure 1:**
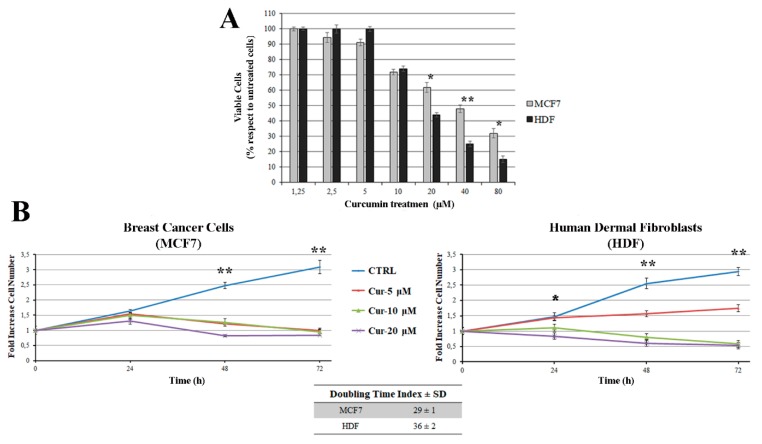
Effect of curcumin treatment on cell growth and cell viability in human dermal fibroblasts (HDFs) and breast cancer cells (MCF7). (**A**) Cell viability was determined by an MTT assay after exposure to an increasing concentration of curcumin for 24 h. (**B**) Cell growth represented as the fold increase in the number of viable cells over 24, 48, and 72 h for HDF and MCF7 cells exposed to 5, 10, and 20 μM of curcumin. The calculated doubling time index is shown in the table. The values in the figures are expressed as the mean ± SD of five independent experiments, each performed in triplicate. A significant difference in cell viability between the two lines was observed in the 20, 40, and 80 μM curcumin treatments. In both cell lines, significant differences in cell growth between the control cells and the curcumin-treated cells were observed (at 48 h and 72 h for all used doses and at 24 h for the HDF cell line in the 10 µM and 20 µM curcumin treatments). * *p* < 0.05 and ** *p* < 0.001.

In order to establish the proper concentration of curcumin to use in the treatment, human dermal fibroblasts (HDFs) and breast cancer cells (MCF7) were exposed to different concentrations of curcumin (0, 2.5, 5, 10, 20, 40, and 80 µM) for 24 h. The results showed a large decrease in cell viability in a dose-dependent manner with different efficacies. At high concentrations (≥20 µM), curcumin treatment induced a decrease in cell viability with respect to the control of 57% and 38% in HDF and MCF7 cells, respectively (Figure 1A).

To investigate the antiproliferative effects induced by curcumin, three concentrations with different degrees of cytotoxicity were selected (not cytotoxic (5 µM), subcytotoxic (10 µM), and cytotoxic (20 µM)). The cells were analyzed at 24, 48, and 72 h after treatment with curcumin. We found that curcumin had a differential ability to inhibit cell proliferation in the two cell lines. A strong inhibition of cell proliferation was observed in HDF cells after 24 h of exposure to 10 µM and 20 µM of curcumin. In MCF7 cells, the antiproliferative effect was found to be delayed: only after 48 h was the cell proliferation inhibited at all tested concentrations (Figure 1B).

In some normal cell lines, such as rat hepatocytes [27], mammary epithelial cells [7], human gingival fibroblasts [28], and human lung epithelial and prostate epithelial cells [29], treatment with curcumin has been shown to be less toxic than in the corresponding cancer cells. Our results show that, at concentrations higher than 10 µM, curcumin induced cell death in normal dermal fibroblast cells. In contrast to our results, some authors have shown that fibroblasts are insensitive to treatment with curcumin. The different findings may be due to the different origins of the studied fibroblasts (neonatal foreskin in [30], fetal lung in [31], and adult skin fibroblasts in our study). Indeed, a number of genes are differentially expressed by epigenetic modifications in the promoter regions. These regulations depend on an external stimulus (such as stress), the tissue’s origin, or temporally programmed activation genes. A highly cytotoxic effect of treatment with curcumin has also been observed in oocytes [18] and immune cells [12,19], increasing potential side-effects on normal cells.

Since the 10 µM curcumin treatment showed a similar cell viability in both cell lines, all subsequent experiments were performed using this concentration in order to explore the mechanisms underlying the antiproliferative action of curcumin in the HDF and MCF7 cell lines.

### 3.2. Curcumin Induces H3 Histone Acetylation and Glutathionylation

**Figure 2:**
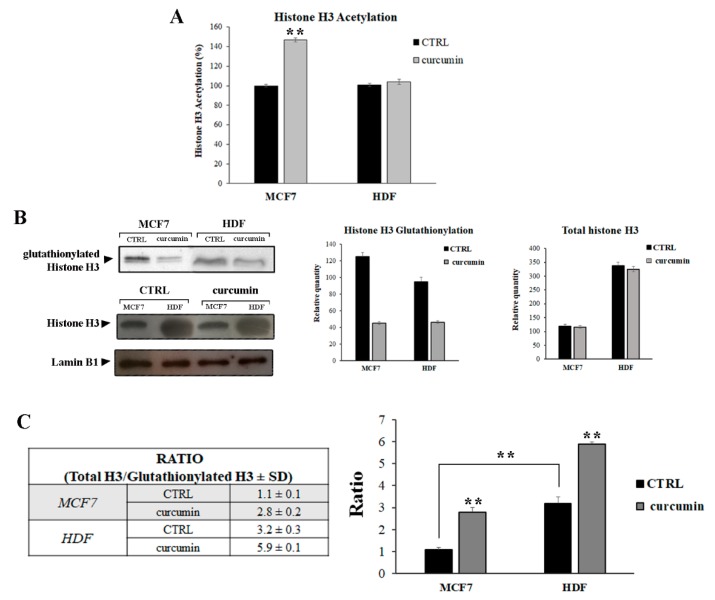
Post-translational modifications (PTMs) of the H3 histone induced by curcumin in MCF7 and HDF cells. (**A**) Histone acetylation evaluated using an acetylation assay kit (Abcam) and analyzed and quantified by ELISA. The percentage of acetylation was calculated as described in the Materials and Methods section. The control for both cell lines was set as 100% histone acetylation. (**B**) Histone S-glutathionylation and total H3 evaluated by Western blotting with anti-glutathione (GSH) and anti-H3 antibodies. The histograms represent the densitometric analysis (*n* = 3). Lamin B1 has been used as a loading control. (**C**) Ratio of total H3 vs. glutathionylated H3. The histogram represents the values reported in the table, calculated by densitometric analysis. ** *p* < 0.001.

Modification of chromatin by PTMs (e.g., acetylation, methylation, phosphorylation, and glutathionylation) plays a key role in the regulation of nuclear processes, including gene transcription, which is fundamental to controlling the cellular metabolism. Covalent modification at the histonic protein level constitutes “the histone code” and, in particular, H3 histone modification has been proposed to play an important role in the control of cell proliferation in mammals [32]. H3 histone variants possess a cysteine whose –SH group may interact with glutathione to form a disulfide bridge that can modify H3 histone interactions and, consequently, modify the degree of chromatin loosening. In this context, we evaluated two PTMs of the H3 histone—“canonical” acetylation and redox-responsive glutathionylation—before and after curcumin treatment. As shown in Figure 2A, treatment with curcumin increased the H3-Ac levels in MCF7 cells by approximately 49% when compared to the untreated cells (CTRL), while in HDF, curcumin did not affect the H3-Ac-level.

Several studies have investigated the effects of curcumin on histone acetylation and deacetylation in tumor cells since irregular histone deacetylase (HDAC) and histone acetyltransferase (HAT) activities were associated with a loss of control over cell proliferation [33]. It has been shown that curcumin is a potent inhibitor of HDAC activity and can lead to an increased level of acetylation, in particular in H4 and H3 histones [34,35,36].

In our study, the upregulation of the acetylated H3 histone after treatment with curcumin in MCF7 cells was probably due to the inhibition of HDACs, as shown by Mukherjee et al. [37]. In HDF cells treated or not treated with curcumin, we did not observe any difference in the H3-Ac levels, which indicates that curcumin did not affect this H3 histone PTM. Apparently, MCF7 and HDF possess different cell-specific targets for the modification of the H3 histone by the action of curcumin.

The H3 histone is the only nucleosomal protein that possesses two cysteine residues, and it can be modified by GSH through S-glutathionylation. Histone glutathionylation affects nucleosome stability, leading the chromatin to have a more relaxed structure. Hence, we evaluated the effect of curcumin on glutathionylation levels of H3 histone in breast cancer and normal dermal fibroblast cells.

The histone fraction was immunoblotted on a PVDF membrane and incubated with an anti-GSH antibody (Figure 2B). Moreover, to evaluate the effective amount of glutathionylated H3 (GLU H3), total H3 analysis in nuclear extracts of both cell lines was performed (Figure 2B). The results show that the H3 histone in untreated cells is more glutathionylated in MCF7 than in HDF. If we consider total H3 vs. GLU H3, we obtain that almost all H3 histone is glutathionylated in MCF7 cells, while in HDF, only about one third is glutathionylated, as shown by the values obtained, indicating a ratio of 1.1 for MCF7 and 3.2 for HDF (Figure 2C, table). These data are in accordance with the study of Garcia-Gimenez et al., in which an increase in H3 histone glutathionylation in cancer cells was observed [38]. In our study, we showed that in untreated MCF7 cells, the doubling time index is lower than HDF cells and, if we relate it with the major H3 glutathionylation level, we can assume that this PTM enhances the speed at which cells proliferate, and that the amount of H3 histone glutathionylation is inversely related to the cellular duplication index.

Garcia-Gimenez et al. observed GSH signals in cancer cell lines but not in confluent human fibroblast cell lines, while we detected GSH signals in HDFs. This divergent result could be due to the fact that our HDF cell line has a confluence of 70% and is not in a confluent status. The detection of GSH signals also confirms that H3 glutathionylation is detectable when a gene transcription is present.

Interestingly, curcumin inhibits H3 histone glutathionylation in both cell lines by three-fold and two-fold in MCF7 and HDF cells, respectively (Figure 2C). These data further consolidate the decrease in speed of cellular proliferation observed after curcumin treatment (Figure 1B).

### 3.3. Curcumin Increases the Total Glutathione Level

Redox homeostasis represents a fine balance between oxidizing and reducing conditions, and its principal marker is the GSH:GSSG ratio [39].

**Figure 3:**
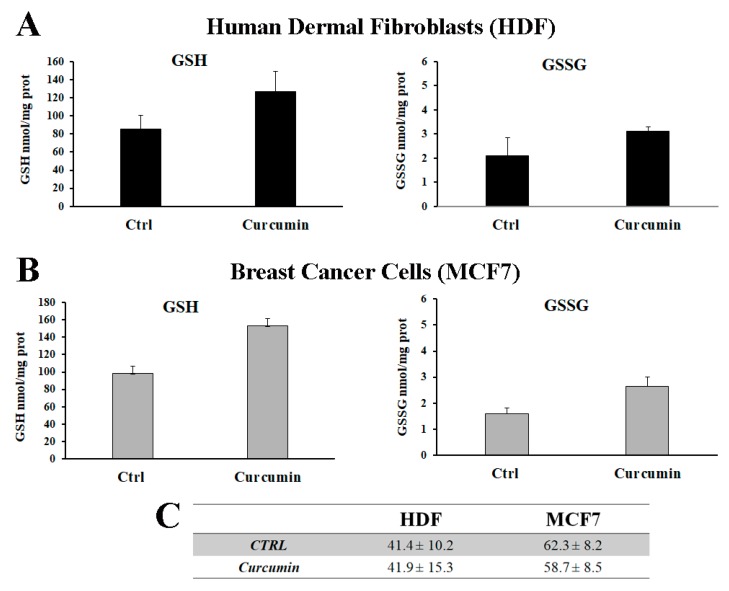
Effect of curcumin on glutathione levels in HDF and MCF7 cell lines. Quantification of total cellular GSH and GSSG of HDF (**A**) and MCF7 cells (**B**). Analysis was performed using a DTNB-glutathione reductase recycling assay and the amount of GSH and GSSG was normalized to protein content. (**C**) GSH:GSSG ratio. Values are expressed as the mean ± SD of six separate experiments.

To determine curcumin’s effect on the redox status of the cells, GSH and GSSG levels were measured after 24 h of treatment. As shown in Figure 3, we observed a significant increase in the GSH level in both MCF7 and HDF cells. This result is in accordance with previous findings that show that curcumin has the ability to induce GSH biosynthesis in Jurkat cells [40] and rat hepatocytes [27]. Curcumin is considered to be a potent antioxidant and a scavenger of a wide range of reactive oxygen species, and these properties could play a role in the maintenance of redox homeostasis [41,42,43].

We observed an increase in the GSSG level in both MCF7 and HDF cells after treatment with curcumin (Figure 3), indicating an active GSH redox cycle [39]. We also observed that the GSH:GSSG ratio remained unchanged in both cell lines after curcumin treatment (Figure 3C) even though the MCF7 and HDF ratio values were different. In this regard, the MCF7 ratio, which was higher than the HDF ratio, is indicative of a more strongly reduced environment in the breast cancer cells.

### 3.4. Curcumin Induces G2/M Cell Cycle Arrest

**Figure 4:**
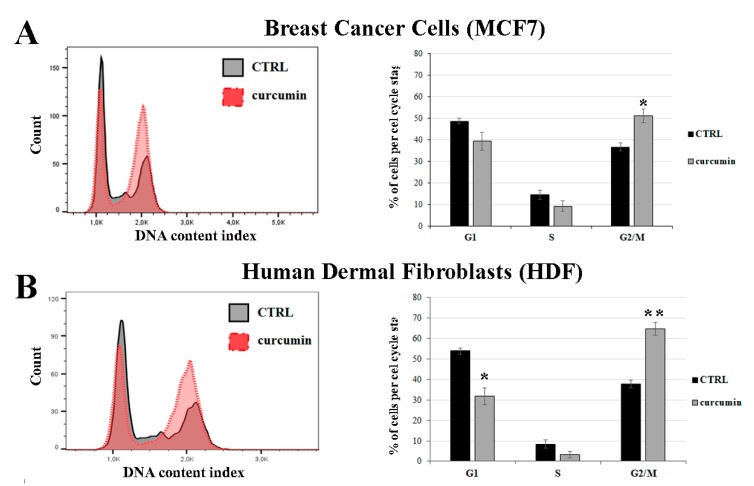
Effect of curcumin on cell cycle progression in MCF7 and HDF cells. (**A**) Breast cancer cells (MCF-7) and (**B**) human dermal fibroblasts (HDFs) treated with 10 µM of curcumin for 24 h and stained with propidium iodide (PI). DNA content was analyzed by flow cytometry. Results are represented as patterns of cells stained with propidium iodide (PI) before and after curcumin treatment (left). The percentage of cell population at different phases (G1, S, and G2/M) of the cell cycle is shown by histograms (right). The images are representative of three separate experiments. * *p* < 0.05 and ** *p* < 0.001 versus an untreated control.

To further evaluate curcumin’s effects on cell proliferation, we performed a cell cycle analysis after 24 h of curcumin treatment in asynchronous cultures. Our results demonstrate that curcumin induced G2/M cell-cycle phase arrest (Figure 4).

The percentage of MCF7 cells in the G2/M phase increased from 37% to 52% after the curcumin treatment (an approximately 1.4-fold increase with respect to the control) (Figure 4A). This result is consistent with previous reports of curcumin inducing G2/M phase arrest in MCF7 cells, which is, in fact, one of the mechanisms underlying the inhibition of cell proliferation in breast cancer [7,13]. Despite this beneficial effect, it is important to note that the G2/M phase arrest occurred at a higher level in HDF cell line, in which we observed an increase in the percentage of cells during the G2/M phase from 38% to approximately 64% (a 1.7-fold increase with respect to untreated cells) (Figure 4B). Therefore, curcumin affects cell cycle progression in MCF7 cancer cells and in normal HDF cells. Since curcumin interacts with several cellular targets, a correlation between the observed PMTs and cell proliferation cannot be clearly delineated; however, the curcumin-induced inhibition of cell growth and decrease in H3 glutathionylation level well correlate with the G2/M cell-cycle phase arrest observed.

### 3.5. Curcumin Induces Necrosis and/or Apoptosis

To determine whether the blockage of normal cell cycle progression involved programmed or nonprogrammed cellular death, apoptosis analysis was performed.

**Figure 5:**
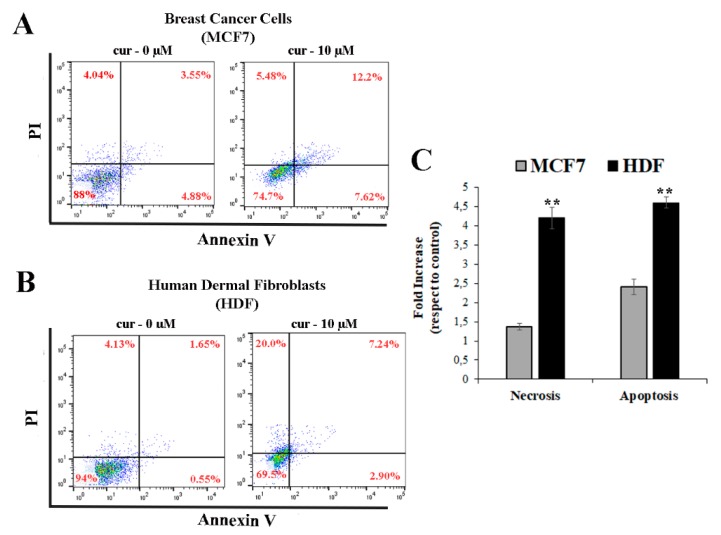
Curcumin-induced apoptosis. HDF and MCF7 cells were treated with 10 μM of curcumin for 24 h followed by washing, trypsinization, and incubation with Annexin V-FITC and propidium iodide (PI). Representative cytograms from three independent experiments are shown for (**A**) MCF7 and (**B**) HDF cells. (**C**) Apoptosis and necrosis quantified as a fold increase with respect to the control (untreated cells). Total apoptosis was calculated by considering early apoptosis (lower right) and late apoptosis (upper right). Data are the mean of at least three experiments, and error bars represent ± SD ** *p* ≤ 0.001 versus control.

Cytometric analysis was used to differentiate between living, early apoptotic, late apoptotic/necrotic, and necrotic cells after staining with Annexin V-FITC and propidium iodide (PI) (Figure 5).

Total apoptosis was calculated by considering early apoptosis (lower right) and late apoptosis (upper right). The results indicate that the 10 µM curcumin treatment promoted cell apoptosis in both cell lines (Figure 5A,B). Figure 5C shows the necrosis and apoptosis levels quantified as a fold increase in the PI- or Annexin-V-positive cells with respect to nontreated cells, respectively. As shown in the MCF7 cells, the amount of apoptosis increased from 8.43% at baseline to 19.82% after curcumin treatment (a 2.4-fold increase with respect to untreated breast cancer cells, *p* < 0.001), while no difference was observed in the necrosis levels, confirming the result that was obtained by Hu et al. [13]. At the same curcumin dose, the programmed cell death in the human dermal fibroblasts increased by approximately 4.6-fold when compared to the control, reflecting that HDFs have a higher sensitivity to curcumin-induced apoptosis than MCF7 cells (Figure 5C). Moreover, Scharstuh et al. have demonstrated that treatment with curcumin induces the death of human gingival [44] and foreskin-derived fibroblast cells [28]. However, in this case, the induction of cell death was selectively apoptosis-like. In contrast, in the HDF cell lines, we observed a higher necrosis level (Figure 5B,C). The percentage of PI-positive cells increased from 4.13% to 20.0% after treatment (a 4.8-fold increase with respect to untreated HDF cells, *p* < 0.001), representing, therefore, an important side-effect of curcumin treatment. Unlike apoptosis, cell death by necrosis frequently triggers inflammatory reactions and, therefore, can potentially induce tissue damage.

The necrosis-and apoptosis-inducing pathways are not completely isolated entities; apoptosis can culminate in secondary necrosis in several cell types [45]. The decision to undergo apoptosis or necrosis depends on the activation of specific expression patterns [46] that are regulated by PTMs. The MCF7 cells showed an increase in histone acetylation (Figure 2A) that could be related to the transcriptional activation of a defined set of genes, resulting in the apoptosis of MCF7 cells as well as cell cycle arrest. No differences in H3 acetylation levels were found in HDF cells, probably because the cellular responses that were observed after treatment with curcumin were not due to this epigenetic modification.

## 4. Conclusions

There is a growing body of evidence that suggests that natural polyphenolic compounds have a dark side. Overall, their effects depend on the concentration, the duration of treatment, and specific cell type [47,48,49,50,51,52]. We demonstrated that normal human dermal fibroblasts are particularly sensitive to curcumin treatment, which induces an arrest of cell cycle progression; moreover, high levels of cell death were observed. The cytotoxic effects that were observed after curcumin treatment may be due to PTMs of histones, which lead to a modulation of gene expression. In particular, we observed a selective increase in H3 histone acetylation levels in the MCF7 cancer cells. The glutathionylation of the H3 histone, which compacts the chromatin structure, was decreased in both cell lines. Since the latest epigenetic changes are linked to the proliferative state of the cells [38], we hypothesized that a reduction in H3 glutathionylation contributes to the inhibition of cellular proliferation through G2/M cell-cycle phase arrest in MCF7 and HDF cells. Thus, curcumin treatment can potentially confer benefits, but high doses may be toxic and thereby establish a double edge in dietary supplements or cancer therapies. A rational and critical use of curcumin is required in order to design a safe and effective treatment.
